# Laparoscopic Subtotal Gastrectomy and Sigmoidectomy Combined With Natural Orifice Specimen Extraction Surgery (NOSES) for Synchronous Gastric Cancer and Sigmoid Colon Cancer: A Case Report

**DOI:** 10.3389/fsurg.2022.907288

**Published:** 2022-06-08

**Authors:** Qingshun Zhu, Lei Yu, Guangxu Zhu, Xuguang Jiao, Bowen Li, Jianjun Qu

**Affiliations:** ^1^ Department of Clinical Medical College, Weifang Medical University, Weifang, China; ^2^ Department of General Surgery, The First Affiliated Hospital of Weifang Medical College, Weifang People’s Hospital, Weifang, China

**Keywords:** gastric cancer, sigmoid colon cancer, natural orifice specimen extraction surgery, laparoscopic surgery, case report

## Abstract

**Background:**

Gastric cancer and colon cancer are rarely seen in clinic, but there are still related reports. For gastric cancer and simultaneous colon cancer, surgical resection is the main treatment. Traditional surgery requires an incision from xiphoid process to pubic symphysis. With the progress of minimally invasive technology, laparoscopic surgery is also used in the treatment of gastric cancer, but also in the abdominal incision to remove specimens and in vitro anastomosis of digestive tract. Taking specimens through the natural cavity as a new surgical method can not only reduce the abdominal incision, but also reduce the occurrence of wound-related complications. Here, we report a patient with gastric cancer and colon cancer who was treated in our hospital.

**Case Summary:**

We report a series of patients with gastric cancer and colon cancer. upper abdominal pain was treated in our hospital for 6 months. electronic gastroscopy showed large irregular ulcers on the lesser curvature of the gastric antrum and biopsy showed poorly differentiated adenocarcinoma of the gastric antrum. The enhanced CT of abdomen and pelvis showed irregular thickening of gastric antrum wall, irregular thickening of sigmoid colon wall and no obvious enlarged lymph nodes around. Further electronic enteroscopy showed that the sigmoid colon showed cauliflower protuberance, the intestinal cavity was slightly narrow, the intestinal wall was stiff, and the biopsy pathology showed moderately differentiated adenocarcinoma of the sigmoid colon. No obvious abnormality was found in serological tumor indexes. We diagnosed gastric cancer with sigmoid colon cancer and the patient received Laparoscopic subtotal gastrectomy and sigmoidectomy combined with natural orifice specimen extraction surgery. At present, 12 months after operation, no clear tumor recurrence was found in the metastasis.

**Conclusion:**

We should improve the understanding of gastric cancer and sigmoid cancer and combine examination with pathology to avoid misdiagnosis as metastatic cancer. Laparoscopic subtotal gastrectomy should be performed for tumors with no serosa invasion, body mass index <30 and tumor diameter <6.5 cm. Sigmoidectomy combined with natural nostril sampling is feasible.

## Introduction

With the popularity of gastroscopy and colonoscopy, gastric cancer and sigmoid colon cancer are often diagnosed at the same time. Surgical resection is still the main treatment method for gastric synchronous sigmoid colon cancer. Studies have shown that laparoscopic surgery is similar to open surgery in terms of safety, efficacy, and completeness of tumor resection (1–3). In recent years, specimen collection through the natural orifice has gradually been used in clinical practice as a new surgical method, which can reduce the incidence of wound-related complications while reducing abdominal incisions (4). For gastric cancer with sigmoid colon cancer, there have been reports of laparoscopic synchronous resection (5), but laparoscopic radical gastrectomy combined with radical sigmoid resection for sigmoid cancer combined with natural orifice specimens for the treatment of gastric cancer with sigmoid colon cancer is rarely reported. Recently, we treated a patient with gastric cancer with sigmoid colon cancer, and performed laparoscopic radical operation for gastric cancer and sigmoid colon cancer with specimens taken from the natural orifice. The report is as follows.

## Case Presentation

### Clinical History and Diagnosis

A 73-year-old male patient was admitted to our hospital for 6 months with epigastric pain. Physical examination revealed a soft abdomen, mild upper abdominal tenderness without rebound tenderness, and no obvious mass palpable throughout the abdomen. There was tympanic sound on percussion, negative mobile dullness, no percussion pain in the liver and kidney areas, and no enlarged lymph nodes were palpated in the bilateral supraclavicular areas. Laboratory tests showed no significant increase in tumor markers. Electronic gastroscopy: Huge irregular ulcers with irregular hyperplasia around the lesser curvature of the gastric antrum, surface erosions, and a little oozing blood were seen (Supplementary Figure S1A). Gastric biopsy pathology: poorly differentiated adenocarcinoma. Electronic colonoscopy showed that the sigmoid colon was 20–26 cm away from the anus with a cauliflower-like bulge, with hyperemia, edema and erosion on the surface, around the circumference, the intestinal lumen was slightly narrow, and the intestinal wall was rigid (Supplementary Figure S1B). Enhanced computed tomography (CT) of the abdomen showed irregular thickening of the gastric antrum wall, enhanced enhancement, no obvious surrounding lymph nodes (Supplementary Figure S1C), and the sigmoid colon had local wall thickness and no obvious surrounding swelling. Lymph nodes (Supplementary Figure S1D). Because the patients and their families requested surgical treatment, they signed the informed consent form for surgery. We conducted a multidisciplinary oncology consultation for the patient. After evaluating the patient’s cardiopulmonary function and no obvious surgical contraindications, we decided to perform Laparoscopic subtotal gastrectomy and sigmoidectomy combined with natural orifice specimen extraction (NOSE) under general anesthesia with tracheal intubation.

### Treatment

After successful anesthesia, take the lithotomy position, routinely sterilize the drape, take an incision of about 1 cm above the umbilicus, incision on the skin and all layers of the abdominal wall, insert a 10 mm trocar, establish a pneumoperitoneum, and the carbon dioxide pneumoperitoneum pressure is 10–12 mmHg, and then enter the abdominal cavity. Microscopic examination: no ascites, no metastasis to liver, peritoneum and pelvis. According to the results of preoperative examination and intraoperative exploration, we decided to perform complete laparoscopic radical resection of gastric cancer and sigmoid colon cancer with specimens taken through the natural orifice. We first underwent subtotal gastrectomy. 12 mm trocar was placed as the main operation hole above the midline of the right clavicle and below the costal edge of the left anterior axillary line, and 5 mm trocar was placed above the midline of the left clavicle and below the costal edge of the right anterior axillary line as an auxiliary operation hole. Distal subtotal gastrectomy and standard lymph node dissection were performed under laparoscopy. Digestive tract reconstruction was performed with Billroth II + Braun anastomosis.

To perform sigmoidectomy, 12 mm trocar was placed as the main operating hole at the medial 2 cm of the right anterior superior iliac spine and 5 mm trocar was placed at the left lower abdominal McDonnell’s point (Supplementary Figure S2). Cut the sigmoid colon at the upper and lower 5 cm of the tumor, Cut open the distal intestine, disinfect with iodophor gauze, and remove the gauze through the rectum to prevent abdominal infection. Put the sterile protective bag into the abdominal cavity from the right 12 mm trocar, put the oval forceps through the anus, pull out one end of the sterile protective bag from the anus, and first put the nail head of the tubular stapler into the abdominal cavity by aseptic protective bag. Then the oval forceps were used to remove the stomach and sigmoid colon specimens successively through the anus. After the specimen was removed, the aseptic protective bag was closed and protruded through the anus (Supplementary Figure S3). Close the broken end of the rectum, insert the nail anvil head of the tubular stapler in the proximal colon by reverse puncture, and insert the tubular anastomosis through the anus for end-to-side anastomosis of the rectosigmoid colon, and the anastomosis can be strengthened with absorbable sutures. The abdominal cavity and pelvic cavity were washed, no active bleeding was examined. Abdominal drainage was placed in the splenic fossa, under the gallbladder and in the pelvis and suture the abdominal incision (Supplementary Figure S4A).

The operation time was 365 min, the blood loss was 50 mL, specimens of subtotal gastrectomy and sigmoid colon in Supplementary Figure S4B. The patient got out of bed 36 h after the operation and was discharged from the hospital 10 days later. Postoperative pathology: (1) Gastric poorly differentiated adenocarcinoma, with an area of 4.5 cm × 3.5 cm, invading the full thickness of the gastric wall, no clear nerve invasion and intravascular tumor thrombus, positive serosal surface, and 48 lymph nodes were detected, none of which were found. See cancer metastasis, no cancer metastasis in omentum tissue, mouth, anus clean margins. (2) Moderately differentiated adenocarcinoma of the sigmoid colon, with an area of 3.5 cm × 2 cm, invading the entire thickness of the intestinal wall and reaching the fat, with tumor thrombi in the vessels, no clear nerve invasion, clean serosa, and 17 lymph nodes were detected around the intestine, all of which were No cancer metastasis was found, the mesenteric resection margin and the surrounding near incision margin were clean, and the mouth and anal incision margins were clean.

### Outcome and Follow-Up

There were no complications after the operation, and the patient was discharged smoothly. As of 12 months after operation, no tumor recurrence or metastasis was found.

## Discussion

Multiple primary cancer (MPC), also known as compound cancer or repeat cancer, refers to the occurrence of two or more independent primary cancers in the same individual at the same time or in succession. We can understand that (1) every tumor is malignant; (2) the tumor is not the recurrence or metastasis of other tumors; for multiple primary cancers of the digestive system, with the popularization of endoscopy and CT examination, its detection The incidence rate continues to increase, and pathological diagnosis is still the gold standard for the diagnosis of multiple primary cancers. In this case, gastric cancer was first diagnosed by electronic gastroscopy, and sigmoid colon was found to occupy the sigmoid colon during preoperative CT examination, and sigmoid colon cancer was confirmed by colonoscopy. It was a double primary cancer of gastric cancer combined with sigmoid colon cancer. Pre-evaluation is particularly important for the diagnosis of multiple primary cancers. If this patient was misdiagnosed as gastric cancer with colonic metastases, the opportunity for surgery may be missed. Although gastric cancer with colon cancer is rare in clinical practice, there are still 1.3%–3.9% of patients with gastric cancer and colon cancer at the same time. Therefore, it is necessary to use colonoscopy as a routine preoperative examination item for gastric cancer (6–8).

In laparoscopic surgery, whether the placement of trocar is reasonable or not is an important factor affecting the operation. The basic principle is that the operation is convenient, the instruments do not interfere with each other, generally the focus as the center into a fan or diamond distribution is the best. In this case, we first selected the umbilical 1 cm to place the first trocar as the observation hole. In gastric surgery, placing the observation hole above the navel can obtain a good surgical field of vision and is more conducive to the clearance of the upper edge of the pancreas. In sigmoid surgery, because the inferior mesenteric blood vessel is located at the umbilical level, if the observation hole is placed in the umbilical part or too close to the operation site, it is easy to cause lens contamination. Placing the observation hole above the umbilical can avoid this problem. At the same time, the auxiliary operation hole during subtotal gastrectomy can be used as an auxiliary operation hole for sigmoidectomy without additional incision, which reduces the postoperative pain and reduces the incidence of postoperative complications such as trocar hole infection and incision hernia.

For gastric cancer with colon cancer, the conventional surgical method requires an incision from the xiphoid process to the pubic symphysis, because such an incision can ensure thorough lymph node dissection at the same time as tumor resection, severe postoperative pain, and many wound complications. With the advancement of minimally invasive technology, laparoscopy can obtain clearer images and dissect more lymph nodes, but for gastric cancer with colon cancer, laparoscopic surgery also requires a small incision in the abdominal wall to facilitate removal of specimens and anastomosis outside the body. In this case, no additional abdominal incision is required, and rectosigmoid anastomosis can be performed through the anus. Yamamot et al. (9) believe that rectosigmoid end-to-side anastomosis is simple and convenient, and does not increase the risk of anastomotic leakage, and the stool is not in the rectosigmoid blind pouch, which indicates that the channel is well preserved. Pironi et al. (10) believe that the use of tubular stapler for colorectal anastomosis is a valuable choice. Ikeda et al. (11) believe that manual suture can increase the strength of the anastomosis and reduce the incidence of anastomotic leakage. The operation of rectosigmoid double purse end-to-side anastomosis is simple and economical, and the anastomosis can be strengthened under laparoscope, but it should be noted that if the distal intestinal tube is too long and thin, it is difficult for the stapler to reach the closed end. Violent operation will cause intestinal wall tear or bleeding, resulting in postoperative anastomotic leakage, abdominal infection and other complications. Before anastomosis, we should dilate the anus properly, and then wipe the tubular stapler with iodophor, which can not only prevent infection but also increase the degree of lubrication. This tip can better deal with this problem. Secondly, after the completion of the anastomosis, it is necessary to check the patency of the distal and proximal intestines.

At present, NOSES is gradually used in clinical practice because of its few wound complications and quick postoperative recovery, especially in sigmoid resection. Sumer et al. (12) reported for the first time NOSES total laparoscopic palliative subtotal gastrectomy with colon cancer. The patient had advanced gastric cancer combined with colon cancer, gastrointestinal bleeding and obstruction occurred before surgery, and died of cachexia 3 months after surgery. After the patient was diagnosed with dual primary cancers, after multidisciplinary oncology consultation, it was decided to undergo complete laparoscopic radical resection of gastric cancer and sigmoid colon cancer with specimens taken through the natural orifice. Laparoscopic surgery on the stomach and colon is performed at the same time, both specimens can be pulled out through the rectum, no additional abdominal incision is required, avoiding incision-related complications (incision infection, fat liquefaction, incisional hernia), reducing pain and bringing better Beauty results, faster return to normal work and life. At present, the patient is still alive and healthy 12 months after the operation, and the weight has increased by 2 Kg compared with that before the operation.

However, not all patients are suitable for NOSES, and Izquierdo et al. (13) suggested that the tumor diameter <6.5 cm and the body mass index <30 could improve the prognosis of patients by implementing NOSES. In addition, the aseptic principle and the tumor-free principle are the basic principles that must be adhered to in all gastrointestinal surgical operations. At present, some scholars worry that NOSES will increase the probability of abdominal infection. In this case, the specimen was first placed in the rectal cavity before the specimen was pulled out through the natural orifice. Put the sterile plastic protective sleeve into the protective sleeve, first put the stapler into the abdominal cavity through the protective sleeve, and then drag the specimen out of the body through the protective sleeve, the tumor tissue does not fall off due to extrusion. Up to now, no tumor recurrence or metastasis has been found in the patient.

## Conclusion

We should improve our understanding of MPC, and combine the examination and pathology to avoid misdiagnosing it as metastatic cancer. Secondly, for tumors that do not invade the serosa, body mass index <30, and tumor diameter <6.5 cm, laparoscopic subtotal gastrectomy should be performed. and sigmoidectomy combined with natural orifice specimen extraction surgery (NOSES) is feasible.

## Data Availability

The original contributions presented in the study are included in the article/Supplementary Material, further inquiries can be directed to the corresponding author.
