# Supplementary material for: Laparoscopic Subtotal Gastrectomy and Sigmoidectomy Combined With Natural Orifice Specimen Extraction Surgery (NOSES) for Synchronous Gastric Cancer and Sigmoid Colon Cancer: A Case Report
Source: Front Surg. 2022 Jun 8;9:907288. doi: 10.3389/fsurg.2022.907288 (PMC9213650; doi:10.3389/fsurg.2022.907288)
Supplement: Supplementary file 1 [file Table_1_v1.docx]

Supplementary Material

## Supplementary Figures

##
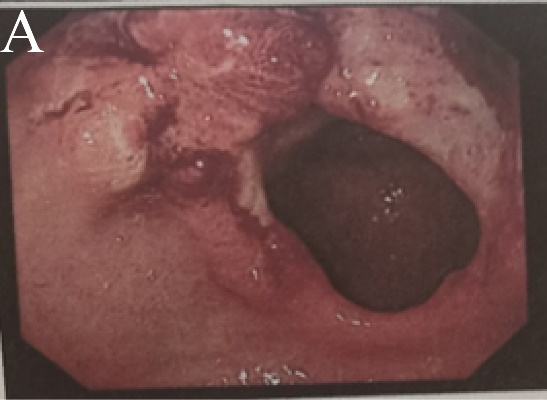

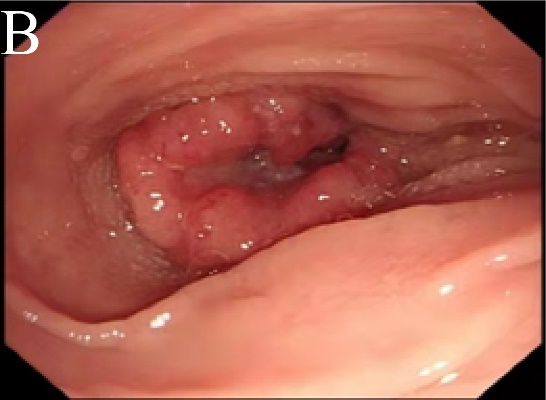


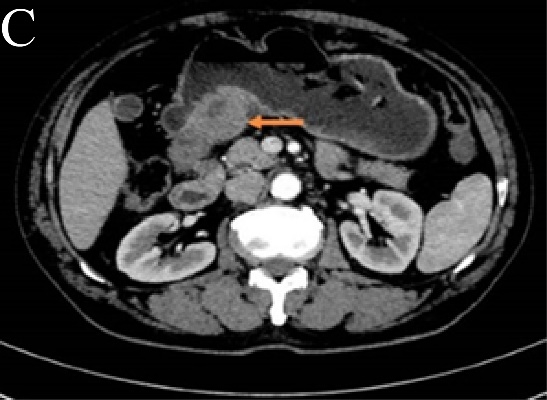

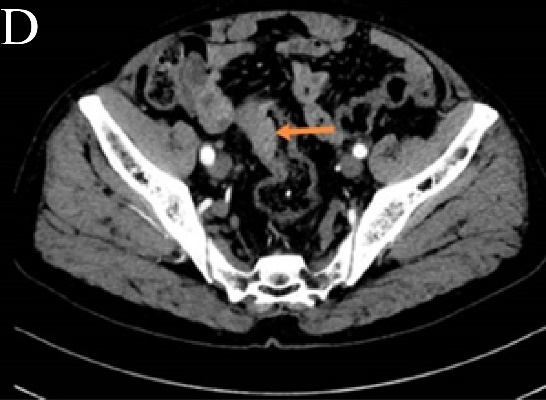


## FIGURE 1 | Elevated space-occupying lesions in the gastric antrum seen by gastroscopy(A). Electronic colonoscopy showed: the sigmoid colon was 20-26cm away from the anus, a cauliflower-like bulge was seen, the surface was congested, edematous, eroded, circumferential, the intestinal lumen was slightly narrow, and the intestinal wall was rigid(B). The arrow on the enhanced CT of the abdomen shows the thickening of the gastric wall in the gastric antrum, and there are no obvious enlarged lymph nodes around(C). The arrow on the enhanced CT of the abdomen shows the thickening of the intestinal wall of the sigmoid colon, and there is no obvious surrounding lymph node enlargement(D).


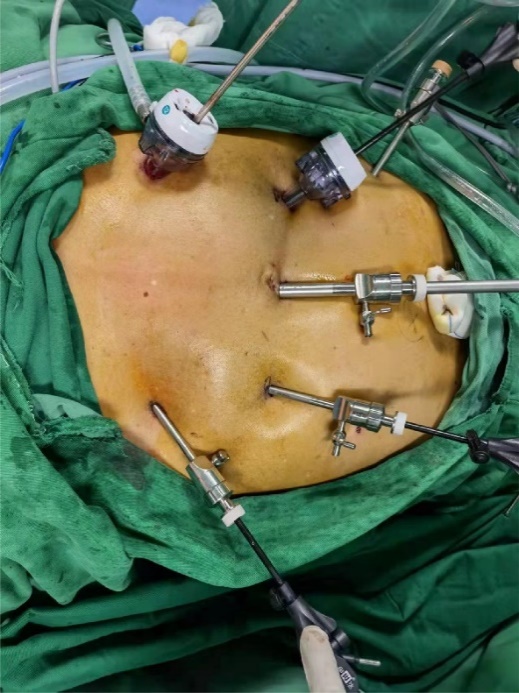


**FIGURE 2** | The location and layout of each trocar during sigmoidectomy. The 12mm trocar in the right abdomen is the main operation hole, and the 5mm trocar in the left abdomen is the auxiliary operation hole. 5mm trocar in the right upper abdomen is an auxiliary operating hole for subtotal gastrectomy.


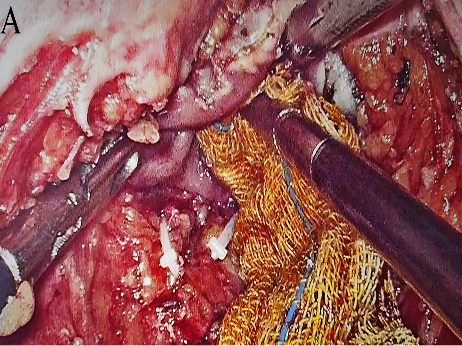

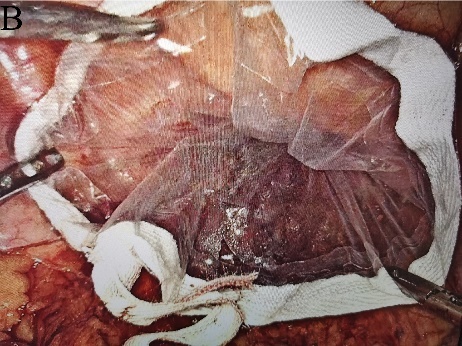

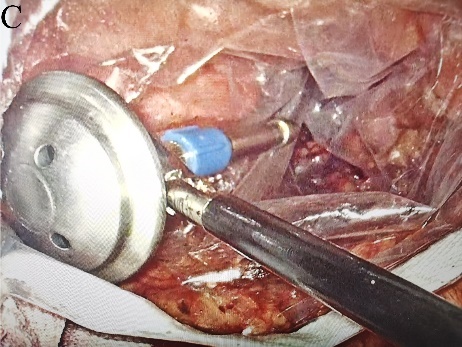


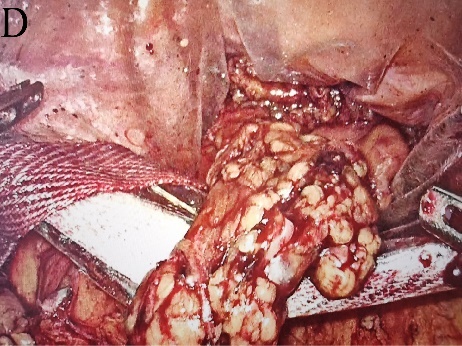

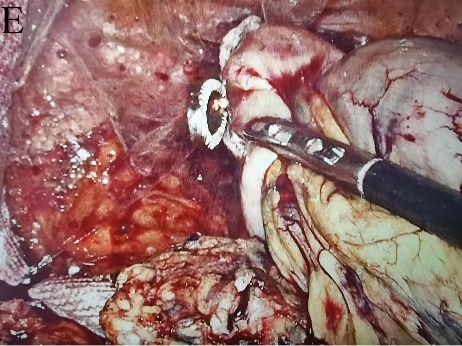

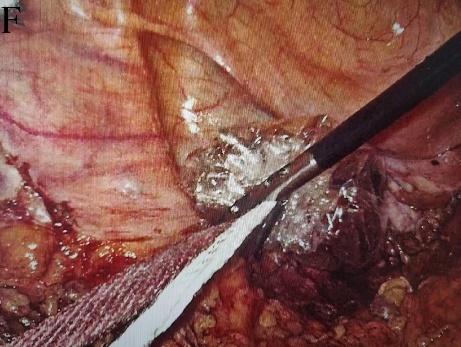


**FIGURE 3** | Cut open the distal intestine and disinfect it with iodophor gauze to prevent abdominal infection(A). The sterile protective bag is placed through the trocar hole, one end is pulled out of the anus with oval forceps, and the other end is unfolded in the abdominal cavity(B). Put the nail anvil head of the tubular stapler into the abdominal cavity through the sterile protective bag(C). Protrude the sigmoid colon from the anus through the sterile protective bag(D). Protrude the distal gastric from the anus through a sterile protective bag(E). After the specimen is removed, the aseptic protective bag is closed and protruded through the anus(F).


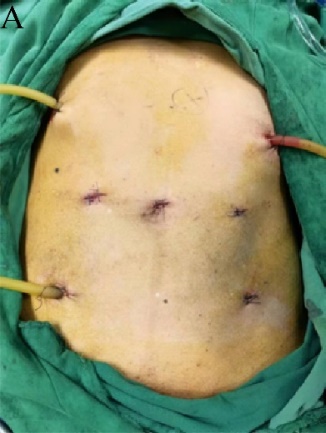

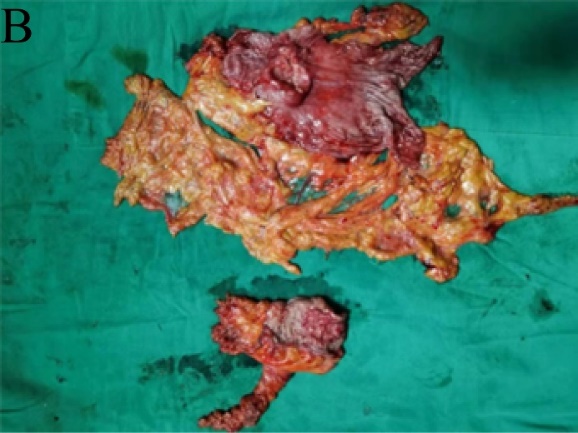


**FIGURE 4** | Location of abdominal incision and drainage tube(A). Specimens of subtotal gastrectomy and sigmoid colon(B).
